# Cognitive Authority Theory: Reframing health inequity, disadvantage and privilege in palliative and end-of-life care

**DOI:** 10.1177/02692163251321713

**Published:** 2025-02-26

**Authors:** Katherine J Hunt, Carl R May

**Affiliations:** 1 School of Health Sciences, University of Southampton, Southampton, UK; 2 Faculty of Public Health and Policy, London School of Hygiene and Tropical Medicine, London, UK

**Keywords:** Theoretical models, palliative care, Cognitive Authority Theory, unconscious bias, social disadvantage in health, health inequalities, health inequities, social privilege

## Abstract

**Background:**

There persist disparities in access to quality palliative and end-of-life care, often based on avoidable injustice. Research and theory to explain this health inequity focuses on structural or individual-based factors, overlooking important relational factors between health professionals, patients and families.

**Aim:**

To apply Cognitive Authority Theory in palliative and end-of-life care to explain neglected relational drivers of inequity in access and experience.

**Methods:**

Cognitive Authority Theory, a middle-range theory of power relations between individuals and authority over knowledge, was developed from empirical and review data. This paper demonstrates its utility in explaining an overlooked component of inequity in palliative care: interactions between health professionals and patients/caregivers.

**Results:**

Using examples from the palliative care literature, we characterise how people who are socially disadvantaged have fewer resources to exploit during consultations with health professionals which makes it difficult for them to have their voices heard, their choices prioritised by others, and to express their expertise. We examine the implications of health professionals’ judgements of expertise for care access, experience, involvement and appropriateness. We offer a fresh perspective on the mechanisms by which stereotypes, bias and power imbalances between health professionals and patients reinforce existing health inequities, drawing on the role of social privilege in shaping inequity in palliative care.

**Conclusion:**

This paper provides a new language to articulate relational drivers of inequity in palliative care. It explains how to use Cognitive Authority Theory to design and interpret research to determine how healthcare interactions reinforce both social privilege and social disadvantage at end-of-life.

**What is already known about the topic?**

- Despite efforts of practice, policy and research, inequity in access to quality palliative and end-of-life care associated with gender, ethnicity, disability and social class continues to persist.
- Underlying this inequity is an intersecting web of social, cultural, economic and political hierarchies which can create structural vulnerability that constrains healthcare access, opportunity, choice and decision-making.
- Palliative care research with people who are disadvantaged is replete with reports of frustration and distress associated with not being listened to or taken seriously by healthcare providers. However, research to understand health inequity often overlooks relational factors between healthcare providers, patients and their families.

**What this paper adds**

- Cognitive Authority Theory describes the relational processes between patients, families and healthcare providers, to explain the mechanisms by which health professional judgement of a patient and family’s competence and expertise (their Cognitive Authority) impacts their involvement in care, the appropriateness of care approaches, and access to other services.
- This paper explains, for the first time, how Cognitive Authority Theory represents a new way of understanding how the consequences of structural vulnerability are represented during clinical interactions with people at the end-of-life or those receiving palliative care, explaining why people who are socially disadvantaged often find it harder to have their voices heard.
- Judgements of cognitive authority can be distorted by (unconscious) bias, helping to explain how intersecting advantages or disadvantages shape access to palliative care services and experience of care at end-of-life.
- Cognitive authority is an important expression of both disadvantage and privilege, and key to understanding how systemic injustices and inequalities are embedded within palliative care and our wider health system.

**Implications for practice, theory or policy**

- Cognitive Authority Theory provides the theoretical underpinning for research to explore and address the unexplored relational drivers of health inequity in palliative and end-of-life care, providing the new terminology we need to expose and describe epistemic injustice associated with a failure to recognise patient and family/caregiver expertise.
- Palliative care research should use Cognitive Authority Theory to unpick interactions between patients, families and health professionals, focusing this not only on people experiencing social disadvantage, but also those experiencing social privilege. This will identify how people use their resources to demonstrate expertise, how health professionals make judgements about the competence of those in their care, and the impact of these judgements for patient involvement and access to palliative care and treatments.
- Although there are important international and cultural differences in decision-making models, preferences for involvement in care, and availability of resources; the desire to be acknowledged and listened to transcends cultural and geographical borders. Cognitive Authority Theory gives us the tools to consider preferences for the recognition and sharing of information and expertise between patients, families and healthcare providers, regardless of cultural norms or preferences.

## Introduction

Structural inequalities in health lead to earlier onset of multiple comorbidities, and earlier death for a substantial proportion of the population.^1–4^ The relationship between Socio-Economic Status (especially employment, income, housing, and education)^5,6^ and health arises from a complex interplay between social and structural factors which create disadvantages that layer in cumulative, intersecting and corrosive ways.^7^ This means that one disadvantage often seeds another,^8^ compounding discrimination, and perpetuating a cycle of inequality^9^ that leads to the production and propagation of inequity (defined as *avoidable* social injustice), associated with gender, ethnicity, sexuality, disability and poverty.^10,11^

The backdrop to this inequity is an interwoven tapestry of social, cultural, economic and political hierarchies that privilege some and subordinate others. Position in these hierarchies shapes susceptibility to harm and social disadvantage, and engenders *structurally induced* vulnerabilities.^12^ This paper is concerned with some of the consequences of these vulnerabilities for people experiencing palliative and end-of-life care and their families. As Stajduhar and Gott^13^ observe:

people who are negatively impacted by often intersecting inequities (e.g. racism, classism, ageism, ableism, sexism) and who experience social disadvantages (e.g. homelessness, poverty), are known to face disproportionate barriers in accessing palliative care.

Despite the continuing efforts of clinicians and some policy-makers, there persist stark inequities in access to palliative and end-of-life care^14–17^ that reflect these structural vulnerabilities.^18,19^ These constrain access, opportunity, choice and decision-making at end-of-life.^15,20^ Thus, people can be created vulnerable by a system that allows assumptions about their identities, social, and living circumstances to shape policy practices and care decisions that lead them to experience exclusion or differential treatment.^19^ This disparity led the Worldwide Hospice Palliative Care Alliance to call for equity-based palliative care and express an urgent need to tackle structural inequities in end-of-life experience.^13^ Yet, there remains a lack of underpinning theory in palliative care research to understand this inequity.^21^

Empirical studies of how institutional power relations shape the experience of people at end-of-life have investigated the ways in which these are shaped by professional communications and patient awareness,^22,23^ as well as their psychological consequences.^24,25^ Despite the person-centredness of these research programmes, studies exploring end-of-life experiences of people who are disadvantaged are thick with accounts of frustration and stress associated with not being listened to or taken seriously by the health professionals who decide on treatments and access to services.^16,26–38^ This suggests a relational component to disadvantage and health inequity where the expertise, or *cognitive authority*, of some people is assumed to be less valuable than others. This paper provides the first account of how Cognitive Authority Theory^39^ can help us understand how structural vulnerabilities shape policy and professionals’ judgements about patients and their families in ways that influence their care, and access to services. These *social* judgements – and the power dynamics that come with them – have important implications for health equity at the end-of-life. In the sections that follow, we use examples from the palliative care literature and two case examples (Figures 3 and 4) to bring life to the theory’s constructs, illuminate previously under-explored drivers of inequity in palliative care, and make recommendations for how to conduct and interpret new research to address inequity.

## Cognitive Authority Theory: Development, meaning and use in health care research

**Figure 1.**
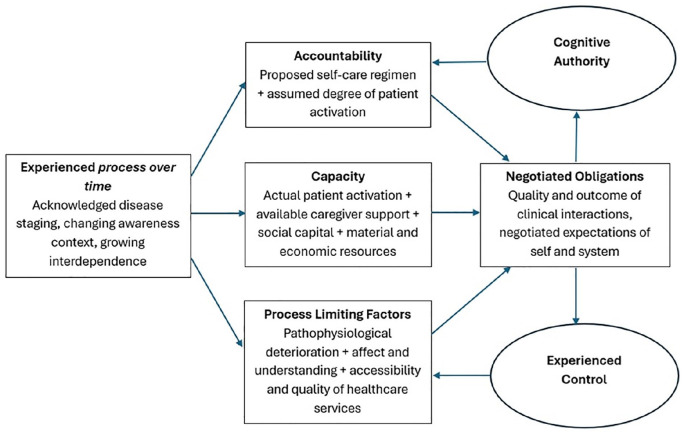
Cognitive Authority Theory.^39^

Cognitive Authority Theory^39^ is a middle-range theory of behavioural and social mechanisms in any setting where inequalities of power exist between actors (Figure 1). It describes human agency under conditions of constraint, explaining how people demonstrate their competence and expertise to perform in the way expected by those with greater degrees of power. Building on a long tradition of research on the lived experience of illness that has focused on the work of being a patient,^40–44^ we developed Cognitive Authority Theory in four stages^39^:

(1) A qualitative elicitation study of people with heart failure was conducted to develop general propositions about patient and caregiver experience, and the influence of care organisation and delivery on that experience.
(2) These propositions were refined and confirmed using a systematic review of qualitative research.
(3) Theoretical propositions and constructs were developed, refined and presented as a logic model consisting of two fundamental theoretical propositions.
(4) A construct validation exercise was undertaken, whereby theory constructs informed reanalysis of a set of systematic reviews.

Our development of Cognitive Authority Theory was in response to increasing policy and practice emphasis on shifting the practical work of self-managing long-term conditions from the clinic to the patient and family/caregiver, acknowledging the moral burden associated with taking *responsibility* for that work.^45–50^ We built the theory on the proposition that patients and caregivers need to use their knowledge and expertise to carry out that *work* in a way that aligns with systemic expectations, and that as they do so they are assessed for competency, trustworthiness and credibility by health professionals who hold a position of higher power and authority. Here, throughout their illness trajectories – what Glaser and Strauss called status passages^43^ and what we refer to in Figure 1 as ‘experienced process over time’ – patients interact with health professionals and draw on their own skills and resources (their *Capacity*) to demonstrate what they know, assert their needs and negotiate what is expected of them by care plans (their *Accountability*). These interactions produce sets of *Negotiated Obligations* which have consequences for

(i) the degree to which health professionals delegate a patient or caregiver/family *Cognitive Authority* (a social judgement of knowledge and competence) over clinical decisions, choices, care and treatments; and
(ii) the extent to which a patient or caregiver/family can *Experience Control* over the structural or resource-based constraints, or institutional rules or biases (*Process Limiting Factors*) that shape their ability to respond to their own needs or carry out the work of treatment.

*Negotiated Obligations* provide the space to manage, through relational negotiation, the limits on *Control* over *Process Limiting Factors* (Table 1). These *Limiting Factors* are external mechanisms that intervene in a way that constrain *Capacity* and *Accountability*, such as deterioration and disease, or larger-scale institutional and structural mechanisms that impact on access to care and treatment, such as resource allocation and service organisation.^46^ Reviews of participation in self-care in heart failure,^51^ chronic obstructive pulmonary disease and lung cancer,^52^ and end-stage kidney disease^53^ found that patients use opportunities to shape *Negotiated Obligations* in ways that allow them to exert *Experienced Control* over access to care and to demonstrate *Cognitive Authority* to make decisions about treatments and advance care planning.

**Table 1.** Definitions of key theory constructs.

Construct	Explanation
Capacity	The material, physical, cognitive, relational, and informational resources that can be mobilised by individuals and groups
Accountability	Normative expectations of patients and caregivers related to their role in their health, care and treatment
Process limiting factors	External factors that challenge a person’s capacity to meet accountabilities
Negotiated obligations	A series of agreed tasks established through discussion and consensus
Cognitive Authority	The product of an assessment of competence, trustworthiness and credibility made about patients and caregivers by other participants in a process (health professionals)
Experienced Control	Extent to which patients and caregivers assess the negotiated obligations assigned to them as practicable *and* the degree to which they successfully manage the external process limiting factors that make it difficult to manage their accountabilities

## Application of Cognitive Authority Theory in palliative care research: How relational processes between healthcare providers and patients perpetuate and sustain inequity

Where patient and caregiver capacity are diminished because of social disadvantage, patients and their families have fewer resources to draw on during interactions with health professionals. This limits their ability to make their voices heard, to have their expertise recognised and to express Cognitive Authority in ways that ensure their choices are prioritised. In turn, they experience inequity through fewer options in care, because of structural or resource-based constraints on *Capacity* (Figures 3 and 4). In McCleskey and Cain’s^31^ study of end-of-life care with diverse populations, people from minoritised ethnic communities experienced this as invalidation of themselves and their caregivers:

Focus on the patient in your face and talk to you in a way that you feel respected and heard. And when your family or loved ones are in the room, pay attention to what they’re saying and not just dismiss them as if they’re irrelevant.

Although white people in that same study reported no concerns about access to good end-of-life care, people from minoritised ethnic communities found that extra efforts were required to access the care they needed:

We got to constantly mandate, monitor, and then challenge. That shouldn’t have to be.^31^

Each person has their own unique pattern of process limiting factors and some, such as racial bias, ageism, and poverty are likely to intersect in ways that disproportionately affect those already disadvantaged. For instance, black women receive poorer quality care at the end-of-life than black men or white women,^33^ socio-economic inequities in access to hospice care are compounded by the location of hospices in more affluent areas^17^; and people living in unfit, poor quality or overcrowded housing may not share the same opportunities to remain at home at the end-of-life as those with greater degrees of privilege.^30^

If done well, palliative care consultations could provide the space for *Negotiated Obligations* to overcome some process limiting factors, and this should be an important focus of work to target discrimination and inequity in palliative and end-of-life care. Indeed, as Stajduhar et al.^16^ found in their work with marginalised and structurally vulnerable people at the end-of-life, referral to palliative care triggered a sense of relief for finally ‘being believed’ by clinicians who seemed invested in their care.

## Addressing inequity in palliative care requires examination of both disadvantage and privilege

Cognitive Authority Theory does not need to explain the causes of health inequity, for that we can turn to Reynold’s Health Power Resources Theory,^54^ and Link and Phelan’s^55^ theory of fundamental causes. It does, however, help us to understand how power imbalances between health professionals, patients and caregivers produce, propagate, and reinforce existing health inequities in palliative care. It can also shed light on how social privilege, an unearned advantage, is reproduced within our health system and revealed by how social position – reflected in identities, behaviours, and vocabularies – influences health professionals’ judgements of the cognitive authority of patients and caregivers during palliative and end-of-life care clinical encounters. Privilege, in this context, reflects:

any entitlement, sanction, power, immunity, and advantage or right granted or conferred by the dominant group to a person or group.^56^

This *entitlement* is important because it implies that people who are socially privileged may *expect* that their cognitive authority will be recognised. This encourages them to take a more active role in palliative care decisions and treatments than people with lesser degrees of privilege and entitlement. In contrast to the experience of people from minoritised ethnic communities in McCleskey and Cain’s^31^ work, participants in Hannon et al.’s^57^ 92% university educated sample of people receiving palliative care found they were able to access whatever they needed:

It’s nice to have a doctor, that if you’re not sure where to go, at least you can go to her or her nurse, they would refer you or do whatever you need to go to the right place to get whatever problem dealt with that you need to have dealt with at the moment.^57^

Unpicking the privilege of Cognitive Authority could bring us closer to understanding how to adapt palliative care encounters to level the playing field than focusing solely on sources and experiences of social disadvantage. However, contemporary approaches to defining and describing health inequity continue to frame it as an unjust disadvantage of some groups rather than an unfair advantage of privileged groups. Here, health services often promulgate a discourse about unequal provision of care that places disadvantaged people at the centre of their own problem,^58^ allowing us to ignore how many of us experience advantage at the expense of others.^13,59–61^

Using Cognitive Authority Theory to disentangle how some people present themselves as more credible in their storytelling, and manage to negotiate expectations, could inform redesign of palliative care consultations in a way that, instead of reinforcing existing inequity, may pose a route to circumnavigating some of the structural sources of inequity. Indeed, people experiencing inequity are likely to have fewer individual and group resources than socially privileged people and thus find it harder to negotiate cognitive authority during healthcare interactions.

## The role of stereotypes and bias in judgement of Cognitive Authority

An important point of exploration is what underpins the judgement of a person’s cognitive authority. While judgement of others is inherent to all human interactions,^62^ negative judgements we make about people who are disadvantaged are learned responses to our immersion in cultural images and narratives that portray certain groups in pejorative and stereotyped ways.^63^ This can be an unconscious, or implicit, bias that occurs without awareness, persists even in equity-minded individuals and impacts perception, memory and behaviour.^64^ Thus, judgement of a person’s expertise and cognitive authority is likely to be biased by stereotypes that reinforce social disadvantage and perpetuate inequity in palliative care:

You pick up a tone, you pick up a, a vibe. I can’t explain it. You see how somebody talks to white people. . . then you, you might ask something similar whatever, and you get a different response, and you might not notice it if you’re the white person. But as black people we do.^27^

Whilst both patients and health professionals bring their biases to the consultation room, research suggests that high levels of health professional implicit bias have a negative impact on important indicators of quality care such as prescribing and patient experience.^63^ Implicit bias inevitably influences health professionals’ assessment of *Cognitive Authority* which can negatively affect patient trust and commitment to treatment.^64^ It can also influence treatment decisions through ‘premature closure’ where clinicians close their mind to options they view as unsuitable^65^ listening only for what they consider medically relevant information. This ‘epistemic injustice’ leads people who are marginalised within our healthcare system to feel that their understanding of their health and illness is discounted by health professionals^66^ and means that other, patient-centred information is often overlooked^67^ resulting in poor care^58^ that is unresponsive to their palliative care needs:

I’m not going to tell them. Because. . . if I start saying I’m a Gypsy. . . I know it’s not going to be the same. So, it’s almost like a self-preservation^28^

Whether it is fair to apportion blame for this state of affairs to health professionals is up for debate, their cognitive authority is also often thrown into doubt through the operation of care pathways, clinical algorithms, and IT systems that elide the social character and lived experience of patients and caregivers. Even so, judgements about a person’s *Cognitive Authority* do illuminate how social determinants of healthcare inequity (ethnicity, gender, class, for instance) are translated into normalised organisational behaviours and are propagated through healthcare structures and social interactions that stratify patients in apparently asocial ways to control access to care. A palliative care patient interviewed by Dewhurst et al.^27^ summarises this articulately:

if you’ve experienced racism within society, why would you then think in, in health that you’re, you know, that you’re going to be treated fairly? And it wouldn’t be obvious. . . but it would be about someone waiting for attention and being overpassed; it would be that subtle.

## Discussion and a call to research

In this paper we have acknowledged three kinds of problem that shape experiences of healthcare at the end-of-life. First, we have pointed to the existence of forms of inequity that when refracted through healthcare services create structural vulnerabilities for patients and caregivers at end-of-life. Second, we have introduced Cognitive Authority Theory as a way of understanding how the consequences of structural vulnerability are represented during clinical interactions, outlining the implications for opportunity, choice and involvement in palliative care. Third, we have discussed the ways that recognised cognitive authority is interwoven with negotiated obligations and distorted by stereotypes and bias. We have focused in on social privilege to examine how intersecting advantages shape access to services and experience of care at end-of-life. In so doing we have shown that cognitive authority is an important expression of both disadvantage and privilege, and key to understanding how systemic injustices and inequalities are embedded within our health system.

As a discipline we must reflect critically on whether palliative care practices and policies address and remedy health inequities or conceal and sustain patterns of healthcare advantage. Models of patient involvement and shared decision-making have brought us closer to recognising the expertise of those our services serve^68,69^ but we must take this work further to address the impact of health professional biases and assumptions on equity of palliative care experience and opportunity. This will require workforce development and training in cultural sensitivity and humility^70^ (in pre-qualifying and postgraduate education), using innovative methods^71,72^ to enable professionals to own their privilege, to be open to their own biases, understand the impact of these on outcomes of interactions with patients, and recognise the advantage afforded to certain groups in clinic and research.^66,73,74^ Cognitive Authority Theory can inform the patient and family involvement agenda, by enabling the understanding we require to facilitate the open and non-judgemental listening and knowledge sharing that patients and their families need to negotiate care that aligns with their priorities, is within their capacity, and supports them to overcome some of the process limiting factors associated with their disadvantage.

**Figure 2.**
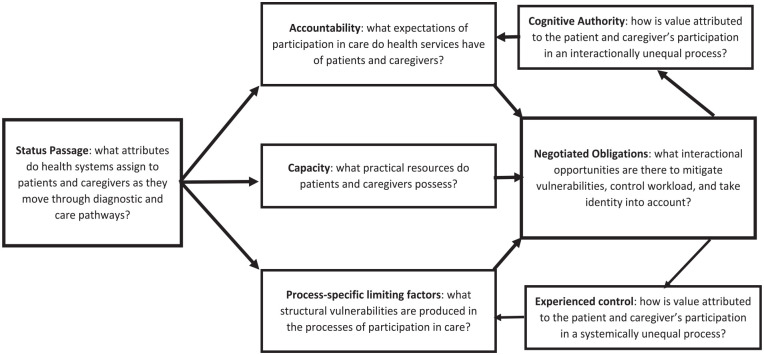
Evidence-based questions.

To do this well we need research that examines what leads us to grant cognitive authority, and the consequences for opportunity, choice and experience. We must also critically analyse the role of those with power and privilege and the way in which health inequities are maintained through their influence on policies and practices that preserve and reproduce that privilege.^58,66,74^ In Figure 2, we show how Cognitive Authority Theory provides an evidence-based set of questions to examine the passage of patients and caregivers through health services marked by the production and reproduction of inequities and vulnerabilities, that have the effect of diminishing their cognitive authority and reducing their experienced control. Addressing these questions will require emancipatory, community-led participatory research using qualitative approaches, to enable understanding of the relational as well as structural and cultural mechanisms involved in the maintenance and propagation of inequity in palliative care.^21^ This should include ethnographic observational methods co-designed and shaped by those experiencing the disadvantage to ensure alertness to the subtle microaggressions familiar to people living with social disadvantage. These observations should focus in on the interactional space between patients, their caregivers and healthcare professionals, in clinic and in the home, to understand more about how Obligations are negotiated and the consequences for Experienced Control and Cognitive Authority over decisions, treatments and care plans. We argue that unearthing the differences in experience between the advantaged and the disadvantaged will shed the greatest light on how to redress the balance. Ergo, observations and qualitative interviews should unpick interactions and delve deeper into unearthed behaviours between disadvantaged as well as privileged people and the professionals they encounter. This must be done with full understanding of the complex power hierarchies that underpin our health services and our wider social system.

An important area of inquiry will be to explore how and why some people with fewer resources and lower capacity still assert their cognitive authority, while others with an abundance of resources do not. Collective social capital, which lies at the heart of building more confident Compassionate Communities, may offer insights here. By involving communities as active participants rather than passive recipients in care; normalizing death, dying, and grieving by fostering death literacy; and recognising that caring for the dying is everyone’s responsibility; compassionate communities build sustainable collective capacity to provide palliative and end-of-life care.^75–77^ Increasing community capacity in this way could support people with fewer individual resources because of social disadvantage to express their Cognitive Authority and experience greater control over access to the care they need. This warrants further study.

**Figure 3.**
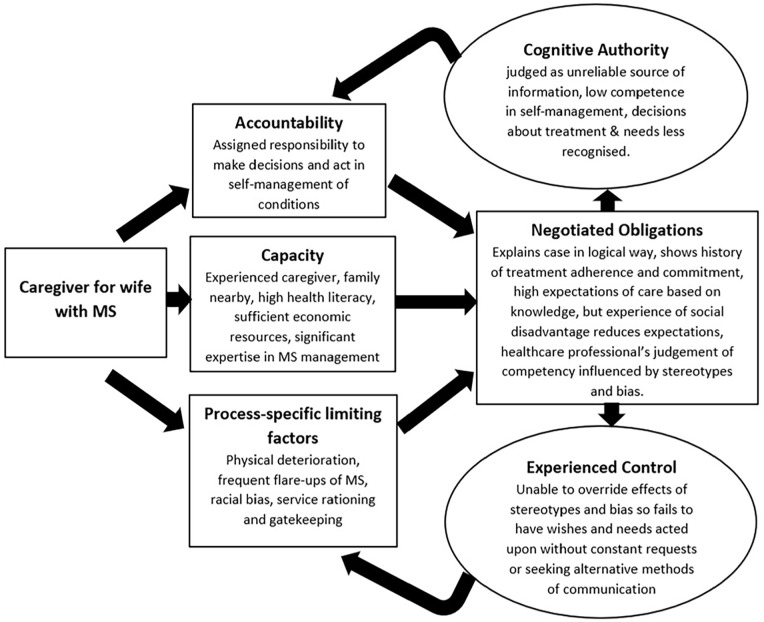
Cognitive Authority Theory, ethnicity and disadvantage. Caregiving case example: Sidney, aged 67, husband to Martha who died from MS. Sidney and Martha had years of caregiving experience, having cared for his mother-in-law until she died. So as Martha deteriorated, Sidney cared for her confidently, supported by their three children (one of whom is a nurse) and their families. Despite his expertise, he didn’t feel listened to during interactions with health professionals. It was hard to explain, but he felt devalued, minimised and judged as having no understanding of health, his wife’s needs, or what she was entitled to. He was inventive and circumnavigated these obstacles by writing to healthcare professionals to negotiate what Martha needed so he couldn’t be dismissed because of the colour of his skin and sound of his voice. Hospital stays were traumatic as there they had no control and felt that no one respected their opinions despite being best placed to explain Martha’s needs. During these admissions, Sidney was a constant presence in the hospital – to provide the diet Martha was used to, as well as feed her and advocate for her as she became more confused towards the end of her life, even though this came at a huge personal and financial cost to him. Martha was offered hospice but chose to die at home, with her family around her and in receipt of community palliative care. The team were wonderful, when they finally got the referral. But looking back, Sidney feels that Martha was shortchanged as they had to fight and constantly ask for things, in ways that made them feel like a nuisance, disempowered and lose faith in the system. They knew what they needed, they understood the signs of repeated infections, flare-ups and decline. But getting health professionals to respond quickly and take them seriously was difficult, and acutely frustrating and distressing.

**Figure 4.**
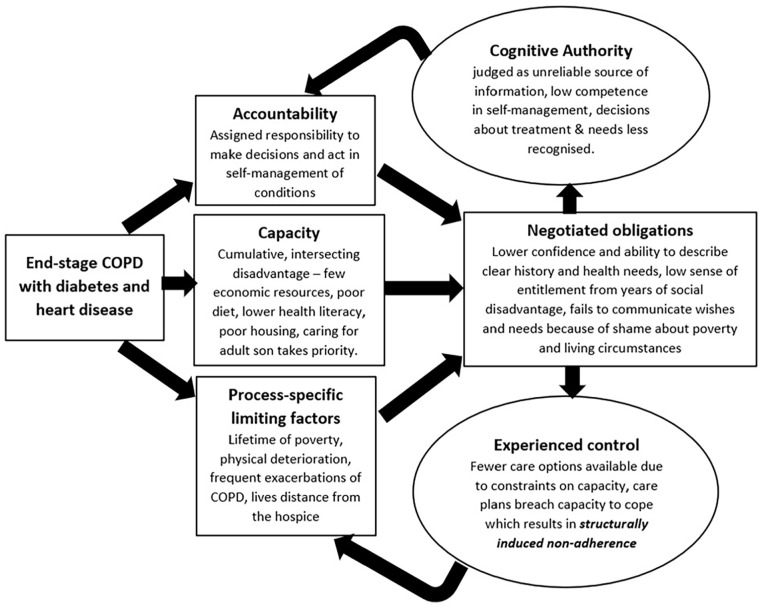
Cognitive Authority Theory and poverty. Patient case example: Beverley, age 59, end-stage COPD with diabetes and heart disease. As Beverley would describe it, she was ‘brought up feral on fish scrapings’ and knew that the poverty and hardship of her childhood had shaped her health. As an adult her living situation continued in the same vein: constant stress from a lifetime of low income and living on the breadline (her husband left years ago) in a damp and mouldy flat surrounded by smoggy roads. In winters she couldn’t afford to heat her house adequately and this exacerbated her COPD. Her adult son had severe mental health problems and lived at home with her, she worried about him and wanted to be there to look after him. Although she was offered care, she didn’t always have the resources to take it up. The physio prescribed a care plan that felt overwhelming, to be honest, and anyway, the travel costs to get there were extortionate so mostly she didn’t attend. She, and all the people she knew, had lived a life of feeling looked down on and treated badly for being ‘poor’. So when her breathing got worse she delayed seeking help, partly because of her son, but also because of shame and fear of judgement from health professionals who came from more financially stable backgrounds and didn’t understand or respect her circumstances. As the exacerbations increased in severity, hospital admissions were inevitable and increasingly frequent. Her anxiety got worse, and the hospital palliative care team became involved. She had an open conversation about her living circumstances with one palliative care nurse, but she didn’t see her again and couldn’t face bringing up the same issues with someone else. They referred her to the hospice day services for ongoing psychological support, but she couldn’t get there without taking two buses, so she declined. Instead, she opted to live day-by-day, and not think too much about what might happen if she got worse.

Application of Cognitive Authority Theory must take account of cultural variation in preferences for involvement in care, decision-making and engagement with healthcare providers; understanding these beyond the confines of Western Individualism.^78,79^ We must do this with recognition that treatment choice and access to palliative care is often a privilege of those living in higher-income countries.^80,81^ While family decision-making is the dominant model in many cultures,^78,82–84^ perception of decision-making varies across regions,^85,86^ and there is international variation in communication styles, hierarchical structures, and prognostic disclosure.^83,87,88^ However, the desire to be listened to and understood by healthcare professionals when sharing medical concerns or histories transcends cultural boundaries and is important for trust, experience and appropriateness of care.^82–84,89^ The World Health Organization’s^90^ Framework on Integrated People-Centred Health Services recognises this, emphasising greater engagement and empowerment of individuals, families and communities. Cognitive Authority Theory gives us the tools to explore how this materialises across regions of the world, to consider reciprocity in knowledge sharing and recognition between patients, families and health professionals; while being open about the impact of power imbalances, cultural norms, and the availability of resources on experience and quality of care (Figures 3 and 4).

## Conclusion

Cognitive Authority Theory can be used to describe the processes involved in the enacting of privilege and advantage in healthcare encounters and interactions. It can explain how some people use their privilege to influence choices available to them, experience greater involvement in decision-making and a more positive and personalised experience of palliative care. It can help explain how health privilege is maintained and perpetuated by describing how the voices and perspectives of privileged people are often seen as more authoritative and legitimate. This means that their narratives and ideas have become the basis for societal norms and health policies, reinforcing existing power structures. Meanwhile, the lived experiences and knowledge of people who are disadvantaged or marginalised are often underestimated or ignored due to systemic biases and stereotypes that perpetuate their marginalization. For the most marginalised in our society this means a double disadvantage: dying younger than their more advantaged counterparts and with potentially avoidable suffering. In this context, we should see acknowledgement of the cognitive authority of the patient and caregiver as a right rather than a privilege – it is much more than recognising that patients and caregivers have values and preferences – but instead calls for investment in dismantling epistemic injustices and fostering supportive and person-centred consultations that level up disparities and support people to demonstrate their cognitive authority. Ensuring that diverse perspectives are incorporated into health policies, practices and research can support our work towards delineating the gap between the moral discourse of promoting equality in access to palliative care, and the actual and unequal distribution of palliative care practices.
